# Intratumoral (Poly-ICLC) Therapy for Dogs with Advanced Cancers: First Report on Clinical Effectiveness, Quality of Life, and Adverse Events

**DOI:** 10.3390/cancers13092237

**Published:** 2021-05-07

**Authors:** Alessandra Rinah Nogueira Voges, Rodrigo Ubukata, Karina Velloso Braga Yazbek, Otávia Luisa Caballero, Andres Mario Salazar, Cristina de Oliveira Massoco, Maria Lucia Zaidan Dagli

**Affiliations:** 1 PROVET, Veterinary Oncology Hospital, São Paulo 04081-004, Brazil; alessandra.rinah@yahoo.com.br (A.R.N.V.); ubukata@gmail.com (R.U.); 2 All Care Vet, São Paulo 04077-003, Brazil; kayazbek@yahoo.com.br; 3 Orygen Biotecnologia, Ltd.a, São Paulo 04544-150, Brazil; ocaballero@orygen.com.br; 4 Oncovir, Inc., Washington, DC 20008, USA; asalazar@oncovir.com; 5 Department of Pathology, School of Veterinary Medicine and Animal Science, University of São Paulo, São Paulo 05508-270, Brazil; cmassoco@usp.br

**Keywords:** cancer, dog, therapy, quality of life, viral RNA

## Abstract

**Simple Summary:**

Although polyinosinic-polycytidylic acid-poly-l-lysine carboxymethylcellulose (poly-ICLC) is widely used as a standalone agent for treating human cancers, there are no reports on its use for treating canine cancers. We aimed to investigate the clinical efficacy, quality of life, and adverse events of poly-ICLC treatment in dogs with advanced cancers. Our results showed that intratumoral poly-ICLC therapy was well tolerated in dogs with advanced cancers, with clinical benefit and improved quality of life scores observed in some dogs. Our findings suggested that patients with lower tumor burden may benefit more from this treatment.

**Abstract:**

Polyinosinic-polycytidylic acid-poly-l-lysine carboxymethylcellulose (poly-ICLC) is a synthetic double-stranded viral RNA analog widely tested as a component of human therapeutic cancer vaccines and as a standalone agent for treating human cancers. However, there are no reports on the use of poly-ICLC for treating canine cancers. This study aimed to investigate the clinical efficacy, quality of life (QL), and adverse events of poly-ICLC treatment in dogs with advanced cancers. The treatment protocol consisted of weekly intratumoral doses of poly-ICLC. The canine patients underwent clinical, laboratory, and imaging tests, and their owners answered weekly QL questionnaires. Fourteen canine patients with different types of spontaneous advanced tumors were enrolled. Most dogs had received prior conventional therapies. Five dogs received at least 12 doses of poly-ICLC: the injected tumor was stable in three dogs, there was a partial response in one, and the injected tumor significantly enlarged in the other. The QL scoring remained stable or increased in most cases. Mild adverse events related to poly-ICLC were observed in 10 of the 14 patients. The data showed that intratumoral poly-ICLC therapy was well tolerated in dogs with advanced cancers, with clinical benefit and improved QL scores observed in some dogs.

## 1. Introduction

Poly-ICLC (polyinosinic-polycytidyl acid, stabilized with poly-L-lysine and carboxymethylcellulose), or Hiltonol^®^ is a synthetic double-stranded viral RNA analog that mimics a danger signal by acting as a pattern recognition receptor agonist [1,2]. Poly-ICLC was initially developed as an interferon inducer, but the current evidence indicates broader biological effects, including specific antitumor and antiviral activities [3,4]. Although Poly-IC and Poly-ICLC have similar effects, as both are recognized by the cytosolic RNA helicase MDA-5 and by TLR3, in order to improve Poly-IC stability to endogenous RNAses in humans, a modified version complexed with poly-Lysine and carboxymethyl cellulose (poly-ICLC) was developed and used in innumerous clinical trials [1,5].

Poly-ICLC activates multiple elements of innate and adaptive immunity, including induction of interferons (IFNs), cytokines and proinflammatory chemokines, maturation of dendritic cells, natural killer cell cytotoxicity, and specific T cell responses, through Toll-like receptor 3 (TLR3) and melanoma differentiation-associated protein 5 (MDA5) [6,7,8]. The exact interaction between dsRNA, IFN, and IFN-inducible systems has not been fully elucidated, but the role of dsRNAs, such as Poly-ICLC may be bimodal: starting with the induction of IFN-related genes and the expression of dsRNA-dependent systems, such as 2′5′OAS, PKR, TLR3, RIG I, MDA5, and probably others, followed by their activation by dsRNA [3,8,9].

Poly-ICLC has been widely tested as a component of therapeutic vaccines for human cancers, including melanoma, gastrointestinal carcinoma, squamous cell carcinoma, gliomas, and multiple myelomas [10,11,12,13,14]. In addition, it has been experimentally employed as a standalone agent for treating established tumors [15,16,17,18,19,20,21].

Cancer is a growing cause of death in canines worldwide [22,23]. Although the prevalence rates of different types of cancer vary, it is estimated that up to 1 in 4 dogs will develop cancer at some point in their life and that almost 50% of dogs over 10 years of age will die from cancer-related problems [23]. The choice of therapeutic protocols for treating canine cancers depends on the histological type and stage of the disease, the general condition of the animal, and the owner’s finances, since many treatments can be very expensive. Standard approaches such as surgery, chemotherapy, and radiotherapy are ineffective in many cases.

The concept of human cancer treatment has changed in recent years, with the immune response’s modulation against tumor cells proving to be an effective therapeutic strategy in an increasing number of cancer types [24,25,26,27]. The field of veterinary cancer immunotherapy has also advanced in the past few decades, but with few approved therapies [28,29]. Nevertheless, cancer immunotherapies are expected to expand the availability of veterinary oncology treatment options in the future.

Poly-ICLC has been investigated in a series of clinical studies in humans, alone or as an adjuvant in cancer vaccines (10–20). As a standalone therapy, when applied intratumorally, the tumor itself is the antigen source, resulting in “autovaccination”. These studies have indicated the clinical safety of using this product in humans, with evidence of efficacy against cancer. Therefore, we investigated whether poly-ICLC treatment could be a therapeutic alternative for canine cancer patients. To our knowledge, there are no previous reports on the use of poly-ICLC for treating canine cancers.

This study aimed to evaluate the clinical efficacy, safety, and quality of life that resulted from intratumoral injections of Poly-ICLC in dogs with advanced cancers.

## 2. Materials and Methods

### 2.1. Patient Eligibility

All the owners of the dogs included in this study signed informed consent forms.

Canine patients with histologically confirmed unresectable tumors of the following types: carcinomas, adenocarcinomas, sarcomas, and lymphomas were considered for the trial. Patients were required to present at least one site with an accessible primary tumor that could be easily injected with poly-ICLC. The lesion had to measure at least 10 mm in the longest diameter and the patient was required to present acceptable renal, hepatic and hematological functions. Owners had to be willing to comply with all the study requirements.

Patients were excluded if they presented, in addition to the tumor, clinically significant unstable medical conditions that could compromise the patient’s safety, severe hepatic impairment, defined as alanine aminotransferase or aspartate aminotransferase > 3 times the upper limit of normal (ULN), total bilirubin > 2 times ULN, or kidney disease classified by the IRIS classification higher than grade II.

### 2.2. Treatment Plan

The study was conducted at the PROVET–Veterinary Oncology Hospital and ALL CARE Vet Hospital in São Paulo, Brazil. As there are no previous reports in the literature of intratumoral Poly-ICLC applications in dogs and taking into account a previous publication of lethality of an intravenous (IV) dose of 1 mg of Poly-ICLC/kg of body weight in 1 of 3 adult dogs [30], the decision was made to use approximately 1/10 of the IV dose for safety reasons. In human clinical trials in which Poly-ICLC was used intratumorally, the frequency of application ranged from one to three times a week [18,20]. We proposed to administer one injection per week to increase compliance with the treatment. The treatment plan consisted of weekly intratumoral injections of poly-ICLC for 12 weeks. Poly-ICLC was administered according to the animal’s weight as follows: animals from 2–5 kg received 0.25 mg, from 5–10 kg received 0.5 mg, and >10 kg received 1 mg. Half of the respective dose was administered in the first week of treatment. If well tolerated, the complete dose was administered between the second and twelfth weeks.

Poly-ICLC was injected at one or more points within a single target lesion throughout the treatment. Analgesics using tramadol hydrochloride and dipyrone were allowed and used as necessary. The use of corticosteroids was avoided as much as possible, unless the inflammatory response was intense and the veterinarian deemed it necessary. No other antitumor therapies were used during the study period. Upon completing the 12-week treatment period, all canine participants continued receiving poly-ICLC on a compassionate basis for as long as the veterinarian judged that the patient benefited from the treatment.

### 2.3. Evaluation Schedule

During screening for inclusion in the study, the canine patients underwent clinical and laboratory evaluations (hematological, blood biochemistry, and urinalysis), a lesion biopsy, tumor staging by ultrasound, radiography, and computed tomography, and immune-inflammatory evaluation (immunophenotyping, complete blood count-based inflammatory score, and immunological evaluation of the tumor biopsy through immunohistochemistry tests). During all visits, immediately prior to the poly-ICLC injection, the patients underwent clinical evaluation and physical examination, including caliper measurement of tumor diameter. In addition, the owners completed the weekly QL questionnaire.

Following the sixth week of treatment and up to ten days after the end of the experimental treatment, the imaging exams and the laboratory evaluations were repeated. A tumor biopsy was taken for histological and immunological evaluation after week 12 of the treatment.

### 2.4. Disease Assessment—Response to Therapy

The response to therapy was determined for the injected (target) lesions and noninjected (or nontarget) lesions. The tumor responses were defined as stable disease (SD), partial response (PR), or complete response (CR) according to the canine response evaluation criteria in solid tumors v1.0 [31]. The tumors were assessed using the one-dimensional measurement of the injected lesion with a caliper. In addition, noninjected lesions were assessed using a computed tomography scan at week 12 of the treatment.

Adverse events were classified according to veterinary cooperative oncology group—common terminology criteria for adverse events (VCOG, 2016) [32]. Toxicity was fully evaluated throughout the study duration and included blood hematologic and biochemical panels, including hematologic, liver, and kidney function.

### 2.5. Quality of Life Scoring

The QL assessment was performed for all dogs during poly-ICLC treatment and consisted of a questionnaire answered by the dog’s owner. The questionnaire consisted of 12 questions with a choice of four answers [33]. Responses to each question ranged from zero to three points, resulting in up to 36 points. A score of zero was considered the worst QL, and 36 was the best. The questions addressed behavior, interaction with the owner, assessment of pain, appetite, sleep disturbances, and signs of vomiting, diarrhea, urinary incontinence, or repletion.

## 3. Results

### 3.1. Patients’ Characteristics

The characteristics of the canine patients are presented in Table 1. Nine females and five males from different breeds (7 Mongrels, 1 Cocker Spaniel, 1 Labrador, 1 Teckel, 1 Maltese, 1 Shih-Tsu, and 1 Bulldog) aged 3–16 years and presenting different types of spontaneous tumors were enrolled in the study.

There were five cases of soft tissue sarcomas, one histiocytic sarcoma, two carcinomas, four adenocarcinomas, and two multicentric non-Hodgkin lymphomas. Most cases were in the World Health Organization (WHO) stage IV (11/14 cases), 2/14 cases were in stage III, and 1/14 cases was stage II. Most tumors (13/14) had metastasized, mostly to the lymph nodes, lungs, liver, and spleen. Prior therapies included surgery (6/14), chemotherapy (8/14), radiotherapy (1/14), and electrochemotherapy (1/14). Two of the 14 cases had no prior treatment.

### 3.2. Response to Therapy

**Figure 1:**
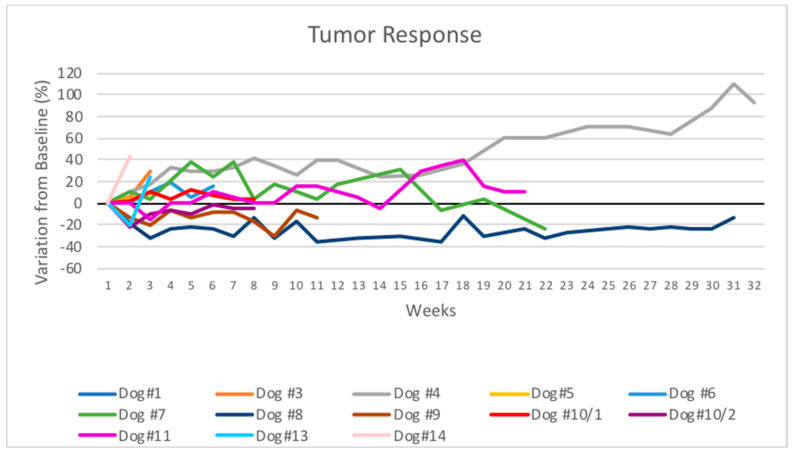
Spider plot of tumor burden changes during intratumoral Poly-ICLC therapy in 12 patients. The longest diameters of the injected lesions are demonstrated as changes from baseline of one lesion from each dog, except from Dog #10 that had two lesions injected with Poly-ICLC.

The target lesions’ responses, injected with Poly-ICLC, are presented in Table 2 and Figure 1.

In the canine patients that completed the course of treatment of 12 weeks or more, 3/5 exhibited less than a 20% increase in the size of the target lesion (SD), 1/5 exhibited at least a 30% reduction in tumor diameter (PR), and one presented more than a 20% increase in tumor size (PD).

Three of the five animals were alive 21 weeks after the start of poly-ICLC treatment, with just one being euthanized because of eventual progressive disease and another animal died due to a preexisting cardiac condition. Thus, in most canine patients that received the full proposed treatment course, a prolonged stabilization of the injected tumor was achieved. An adenocarcinoma patient that left the study after eight weeks for reasons unrelated to the disease (dog 10) exhibited stability of the injected lesion confirmed by CT measurement at week nine. The majority of cases received less than the full course of treatment due to either progressive disease or other issues that precluded continuation in the study. The three patients that presented stages II and III (dogs 4, 7, and 8) had the best outcomes in the study. The disease remained stable in patient 4 for 12 weeks before progressing. Patient 7 is still being treated on a compassionate basis, and although the tumor increased in size in the first month of treatment, it remained practically stable for the following five weeks before starting to decrease. Patient 8 presented PR in the injected lesion and died of diseases unrelated to the tumor 31 weeks after starting the treatment.

The remaining patients that were treated with poly-ICLC for six weeks or less had progressive disease (6/8 cases). In these cases, the treatment was generally unable to restrict tumor growth, possibly due to its more advanced stage at the start of treatment. Notably, the initial size of the injected tumors of those patients that were unable to complete the full course of treatment were, in all cases except dog 13, more than twice as large as the tumors of the patients that were able to complete the treatment. The baseline mean of the size of the injected tumors in the dogs that received the full course of treatment was 3.64 cm, whereas in the dogs that received six or fewer injections, the mean baseline tumor size was 9.86 cm.

For quantifying the effects of poly-ICLC on nontarget lesions, five patients that had at least two measurements of the lesions using computed tomography were analyzed (Table 3). Most metastatic lesions increased in size, and new metastases were identified in three of the five cases. Overall, the nontarget lesions progressed in four patients, whereas one patient presented SD. Thus, even in the cases where there was an apparent control of the target tumor, there was no abscopal effect.

### 3.3. Quality of Life Scoring

**Figure 2:**
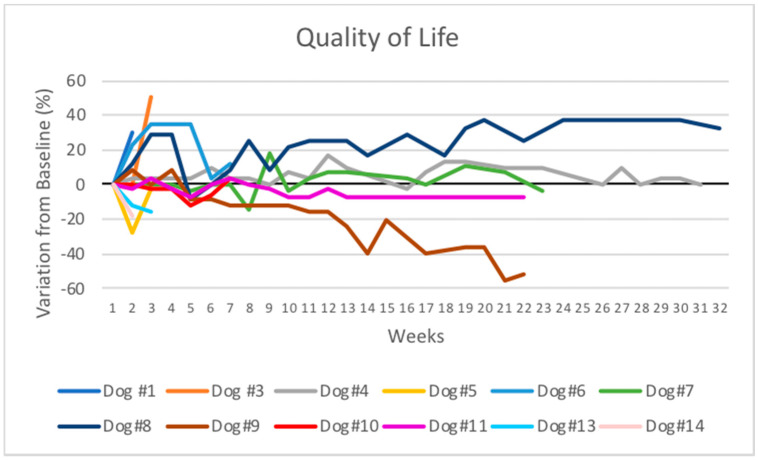
Spider plot of quality of life scores during intratumoral poly-ICLC therapy in 12 patients. The quality of life scores are demonstrated as changes from baseline.

The variation of the QL scores in relation to the baseline measurements of canine patients that received one, two, or more poly-ICLC injections are presented in Figure 2. The QL score remained stable during the treatment for most dogs. The only dog that presented partial tumor response (dog 8) presented an increase in QL scores throughout the treatment period that varied from 8% to 37% in relation to the baseline measurement. Before starting the treatment, this dog had difficulties defecating, probably due to the perianal lesion and lymph node enlargements, which resolved after three doses of poly-ICLC. In this patient, analgesics could be reduced and were removed from the treatment protocol after nine weeks of treatment with poly-ICLC.

One patient (dog 9) presented a marked decrease in QL scores that reached 56% at week 21. This patient presented a very large subcutaneous lesion that evolved with an important inflammation of the subcutaneous tissue of the caudal and ventral abdominal region and edema of the hind paw. Treatment with corticosteroids decreased the inflammation, but the disease progressed with many nodules in the inguinal and the inner face of the right pelvic limb. The patient was euthanized 181 days after the initiation of treatment due to progressive disease and marked QL decrease.

**Figure 3:**
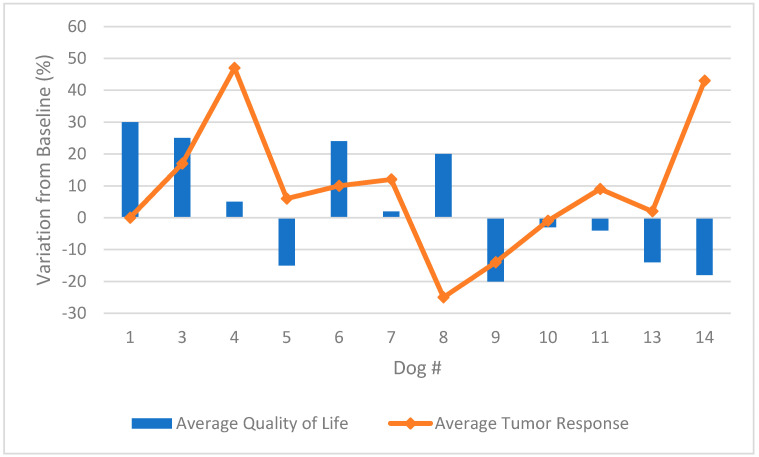
Combination chart of the average change from baseline QL scores and tumor response scores during intratumoral poly-ICLC therapy in 12 patients.

A combination chart with quality of life and tumor response is shown in Figure 3. There was no obvious correlation between measured tumor response and quality of life.

### 3.4. Adverse Events

The adverse events related and unrelated to poly-ICLC treatment are presented in Table 4. All 14 dogs that received at least one intratumoral dose of poly-ICLC were included in this assessment. Adverse events related to poly-ICLC were mild and included grade 1 lethargy and fatigue in half of the dogs. Allergic/hypersensitivity reactions were observed in three dogs: one had a grade 1 reaction, and two had grade 2 reactions. There was no discontinuation of the treatment due to adverse events.

## 4. Discussion

This study represents the first report on the use of poly-ICLC for treating canine cancers, in which a strategy of therapeutic intratumoral injections of the dsRNA viral mimic and TLR agonist, poly-ICLC was tested. This strategy was well tolerated and generated clinical benefit in some patients.

There is considerable interest in developing immunotherapies for canine cancers [34,35,36]. Poly-ICLC represents a highly practical potential option for this aim. Poly-ICLC, a synthetic complex of carboxymethylcellulose, polyinosinic-polycytidylic acid, and poly-L-lysine, can stimulate cytotoxic cytokine release and increase the tumoricidal activities of various immunohematopoietic cells by inducing interferon-gamma production [18]. This phenomenon has been extensively investigated in humans as a standalone therapy or combined with vaccine antigens.

Salazar and colleagues [15] first investigated in 1996 the use of poly-ICLC for the long-term treatment of human cancers at a dose similar to that used in our study. This was a pilot study for the treatment of malignant gliomas and showed prolonged quality of survival with tumor stabilization or regression. Several trials have been conducted since then, including poly-ICLC as the therapeutic immunostimulant for different types of human cancers (10–20).

The present trial involved 14 dogs, of which five received the planned 12 weekly doses of poly-ICLC. Three of these dogs presented stable disease, and one showed partial response. Notably, the quality of life of the patients receiving long-term poly-ICLC remained stable or improved throughout the planned treatment.

The owners reported important improvements in the quality of life of their dogs. Considering the advanced neoplasm of these animals, we consider the achievement of at least three months of stable disease with a good quality of life to be clinically relevant, although randomized placebo control studies would be required to confirm this clinical effect. The best outcome shown in the three patients that presented less advanced disease (WHO stages II and III) may signify that patients with lower tumor burden may receive increased benefit from this treatment.

Nine animals received from one to six doses of poly-ICLC due to their poor clinical condition in most cases. All nine patients were in WHO stage IV and had metastasis.

All 14 dogs included in our study were evaluated for adverse events. Regardless of the type of cancer, the WHO stage, and number of doses received, the adverse events of intratumoral poly-ICLC injections were mild and consisted only of lethargy/fatigue similar to that described in human studies [18]. Low-grade allergy/hypersensitivity reactions were observed in two animals.

Given the advanced stage of the canine patients’ tumors included in our study and the multiple mechanisms for immune suppression in oncologic patients, using a single immune stimulator may not be enough for inducing robust anticancer immunity. Recently, Kyi et al. [18] tested poly-ICLC in patients with recurrent metastatic disease therapy (head and neck squamous cell cancer and melanoma) in which prior systemic treatment had failed. Patients received two intratumoral treatment cycles, followed by intramuscular boosters biweekly for seven weeks, with a one-week rest period. One patient completed two IT treatment cycles and achieved clinical benefit (SD and progression-free survival of six months). Poly-ICLC was well tolerated in patients with solid cancer and generated local and systemic immune responses, as evident in the patient achieving clinical benefit. These results were similar to those obtained in our study.

In a recent study [37], it was shown that intratumoral injection of poly-ICLC was significantly less effective in inducing tumor T cell infiltration and controlling growth of tumors in mice as compared with systemic (intravenous or intramuscular) administration. Systemically administered Poly-ICLC resulted in the enhancement of T cell infiltrates into solid mouse tumors and correlated with a substantial therapeutic effect. Intramuscular or combined IT and IM routes Poly-ICLC treatment may increase the therapeutic benefit in these patients.

## 5. Conclusions

The goal of any trial to test new cancer therapies is to show that its use can improve the patient’s condition without causing adverse events. Overall, it can be concluded from our initial study that poly-ICLC is well tolerated in dogs with different types of advanced cancers, exhibits clinical efficacy in injected lesions and improves the quality of life in some cases. This justifies further trials of poly-ICLC for verifying its benefits for dogs with specific cancers and the inclusion of intramuscular application to achieve an enhanced systemic response.

## Figures and Tables

**Table 1:** Characteristics of the canine patients.

Dog	Breed	Age (Years)	Gender	Primary Tumor	Location	TNM	WHO Stage	Metastasis	Prior Therapies
1	Mongrel	16	Male *	STS	Jaw	T3N1M1	IV	Olfactory bulb	Surgery, chemotherapy (4 Dx)
2	Mongrel	3	Male *	STS	Thorax	T4N1M1	IV	LN, lungs	None
3	Cocker spaniel	13	Female *	STS	Abdominal region	T4N1M1	IV	LN, lungs, liver, spleen	Surgery, ECT
4	Mongrel	8	Female *	STS	Mandible	T3N1M0	III	LN	Surgery, chemotherapy (5 Dx)
5	Mongrel	16	Female *	STS	Thorax	T4N1M1	IV	Lungs, liver	None
6	Labrador	11	Female	Histiocytic sarcoma	Thorax	T3N1M1	IV	LN, lungs, skin, liver, spleen, intestinal serosa, myocardium, esophagus	None
7	Teckel	12	Male *	Carcinoma	Dorsal to the left eye	T2N0M0	II	none	Radiotherapy
8	Maltese	13	Female *	Carcinoma	Left perianal region	T3N1M0	III	LN	Chemotherapy) (RTKi)
9	Mongrel	15	Female *	Adenocarcinoma	Breast, inguinal region and inner face of the right pelvic limb	T4N2M1	IV	LN, skin, liver	Surgery, chemotherapy (4 Dx, 1 Cb)
10	Mongrel	12	Female *	Adenocarcinoma	Paravertebral	T3N2M1	IV	Subcutaneous, lungs	None
11	Shih-tzu	15	Female *	Adenocarcinoma	Breast, vulva	T3N1M1	IV	LN, liver, skin	Surgery, chemotherapy (4 Cb)
12	Mongrel	9	Female *	Adenocarcinoma	Breast	T4N2M1	IV	LN, lungs, skin, liver, spleen	Surgery, chemotherapy (5 Dx, 6 Cb)
13	Bulldog	11	Male *	Multicentric NHL	Lymphoid tissue	stage IV	IV	Liver, Spleen	Chemotherapy (Vc, Cc, Dx, Pr)
14	Mongrel	10	Male *	Multicentric NHL	Lymphoid tissue	stage IV	IV	Liver, Spleen	Chemotherapy (1 Vc, 1 Cc, 1 Dx)

^1^ STS, soft tissue sarcoma; NHL, non-Hodgkin’s lymphoma; LN, lymph node; TNM (Tumor, Lymph node, and Metastasis); WHO, World Health Organization. ECT, eletrochemotherapy; Dx, doxorubicin; Cb, carboplatin; Vc, Vincristine; Cc, cyclophosphamide; RTKi, toceranib (receptor tyrosine kinase inhibitor). * Castrated/Spayed.

**Table 2:** Response to treatment of target lesions injected with poly-ICLC. Dogs were separated according to the number of weeks of treatment.

Dog	Treatment Duration (Weeks)	Dose of poly-ICLC (mg)	BOR	Survival (Days)	Survival after poly-ICLC (Days)	Concurrent Disease	Status
4	30	1.00	PD	411	259	None	alive
7	22	0.50	SD	346	206	MVI	alive
8	31	0.25	PR	520	252	CKD, MVI, TVI	dead (kidney and heart failure)
9	21	1.00	SD	470	181	None	Dead (euthanasia)
11	21	0.50	SD	2108	178	MVI, TVI	alive
10	8	1.00	SD	123	133	None	dead (euthanasia)
6	6	1.00	PD	133	112	None	dead (euthanasia)
5	3	0.50	NE	555	77	MVI	alive
13	3	1.00	PD	218	24	MVI, TVI	Dead (respiratory failure)
1	2	0.50	NE	607	46	MVI, TVI	Dead (seizure)
14	2	1.00	PD	100	24	None	Dead (respiratory failure)
2	1	0.25	PD	127	13	None	Dead (probable cause?)
12	1	0.25	PD	443	6	MVI	Dead (euthanasia)

BOR, Best Overall Response; SD, stable disease; PD, progressive disease; CKD, chronic kidney disease; MVI, mitral valve insufficiency; TVI, tricuspid valve insufficiency; NE, Not Evaluable. According to RECIST (Response Evaluation Criteria in Solid Tumours), a requirement for SD is that it should be met at least once no less than 6–8 weeks after the first dose of trial treatment/baseline assessment, otherwise the best response will be Not Evaluable (NE).

**Table 3:** Response to treatment in nontarget lesions, not injected with poly-ICLC.

Dog	Treatment Duration (Weeks)	Baseline Tumor Burden Nontarget Lesions	Final Tumor Burden Nontarget Lesions	BOR Nontarget Lesions	BOR Target and Nontarget Lesions
4	30	LN: 1 enlarged (3.8 cm)	LN: 1 enlarged (3.9 cm)	SD	SD
8	31	LN: 2 enlarged (4.6 cm and 1.9 cm)	LN: 3 enlarged (4.5, 2.1, 1.4 cm)	PD	PD
9	21	Liver: 2 nodules (4.6 cm and 2.9 cm)	Liver: 2 nodules (4.7 and 3,3 cm) + another Spleen: 2 nodules (0.9 and 2.2 cm)	PD	PD
10	6	Lungs: unquantifiable nodules	Lungs: unquantifiable nodules	PD	PD
11	21	Liver: 1 nodule (5.1 cm) LN: 3 enlarged (1.7, 0.9, 0.5 cm)	Liver: 4 nodules (7.0, 1.0, 0.3, 0.7 cm) LNs: 4 enlarged (2.1, 1.0, 1.5, 1.2 cm) Stomach: 1 nodule (1.2 cm)	PD	PD

BOR, best overall response; SD, stable disease; PD, progressive disease; LN, lymph nodes.

**Table 4:** Adverse events related and not related to poly-ICLC treatment.

Adverse Events	Grade 1	Grade 2	Grade 3	Grade 4	Grade 5	TOTAL
**Related to poly-ICLC**						
Lethargy/fatigue	7/14	0	0	0	0	7/14
Allergic reaction/ hypersensitivity	1/14	2/14	0	0	0	3/14
**Not related to poly-ICLC**						
Skin ulceration	0	2/14	2/14	0	0	4/14
Seizure	0	0	0	1/14	1/14	2/14
Dyspnea	0	0	0	0	3/14	3/14
Increased alkaline phosphatase	1/6	2/6	2/6	0	0	5/6
Increased ALT	0	2/6	2/6	0	0	4/6
Increased BUN	2/6	1/6	0	0	0	3/6
Increased creatinine	1/6	0	0	0	0	1/6
Decreased hemoglobin	5/6	1/6	0	0	0	6/6
Cystitis	1/6	0	0	0	0	1/6

## Data Availability

Data are available upon request.
